# The effects of pedometer-based exercise on central and peripheral vascular functions among young sedentary men with CVD risk factors

**DOI:** 10.3389/fphys.2023.1062751

**Published:** 2023-03-28

**Authors:** Norsuhana Omar, Boon Seng Yeoh, Kalaivani Chellappan, Sara Zijiun Chui, Norizam Salamt, Amilia Aminuddin

**Affiliations:** ^1^ Department of Physiology, School of Medical Sciences, Universiti Sains Malaysia, Kelantan, Malaysia; ^2^ Department of Electrical, Electronic and Systems Engineering, Faculty of Engineering and Built Environment, Universiti Kebangsaan Malaysia, Bangi, Selangor, Malaysia; ^3^ Department of Physiology, Faculty of Medicine, Universiti Kebangsaan Malaysia Medical Centre, Kuala Lumpur, Malaysia

**Keywords:** exercise, finger photoplethysmography, pedometer, cardiovascular, pulse wave velocity, augmentation index

## Abstract

**Introduction:** Cardiovascular diseases (CVDs) remain the main cause of morbidity and mortality in Malaysia and worldwide. This is mainly due to an increase in the prevalence of CVD risk factors such as hypertension, dyslipidemia, smoking, and obesity. Increased physical activity has been recommended as a modality to improve CVD risk. Pulse wave velocity (PWV_CF_), augmentation index (AI), and finger photoplethysmography fitness (PPGF) index have been introduced to assess the vascular functions related to CVD risk factors. The effects of long-term exercise on PPGF index are not established.

**Materials and Methods:** A total of 70 young men who were sedentary with two or more cardiovascular risk factors were recruited. Subjects were randomly assigned to a control group (CG) (n = 34; no change in walking) and pedometer group (PG) (n = 36; minimum target: 8,000 steps/day). PWV_CF_ and AI were measured *via* the Vicorder system. The PPGF index was obtained *via* the finger photoplethysmography method. All parameters were measured at baseline and after 6 and 12 weeks.

**Results:** After intervention, the PG had significant increased step count from 4,996 ± 805 to 10,128 ± 511 steps/day (*p* < 0.001). The PG showed significant improvement in anthropometric variables, lipid, PWV_CF_, AI, and PPGF index (time and group effect *p* < 0.001). No changes were observed in CG.

**Conclusion:** This signifies that pedometer-based walking program is beneficial in improving markers of vascular functions among young working sedentary men with CVD risk factors. Pedometer-based exercise should be encouraged to improve cardiovascular health.

## 1 Introduction

Aerobic exercise has been recognized as a modality for improving cardiovascular health (Wilmore and Knuttgen, 2003). This type of exercise is beneficial in improving hypertension, obesity, diabetes mellitus, dyslipidemia, and many more, which could serve as preventive and therapeutic measures in lowering the CVD risk (Aminuddin et al., 2011; Miele and Headley, 2017; Wewege et al., 2018). Recent guidelines recommended that adults should engage in 150 min per week of accumulated moderate-intensity or 75 min per week of vigorous-intensity aerobic physical activity to reduce the CVD risk (Arnett et al., 2019).

CVD risk factors lead to vascular dysfunction *via* several mechanisms such endothelial dysfunction, increased inflammation, increased oxidative stress, and insulin resistance, leading to fat accumulation in the arterial wall and atherosclerosis (Kattoor et al., 2017; Di Pino and Defronzo, 2019; Milutinović et al., 2020; Mokgalaboni et al., 2020; Monserrat-mesquida et al., 2020; Villano et al., 2020). Monitoring of vascular function is necessary in assessing the severity of the diseases so that management can be initiated at an early phase. There are several methods to monitor vascular function non-invasively, such as flow-mediated dilation, augmentation index, pulse wave velocity, central BP (Tomiyama and Yamashina, 2010), and finger photoplethysmography (PPG).

Pulse wave velocity and augmentation index are among parameters that represent central vascular markers and found to be increased in aortic stiffness (AS). As the heart contracts, the pressure that is generated travels along the aorta and reaches the peripheral artery. If the stiffness of the aorta increases, the aortic pressure (central blood pressure) will increase, and at the same time, the pulse wave pressure travels faster (Aminuddin et al., 2014). Part of the pressure wave is reflected back to the heart and augments the aortic pressure. Carotid femoral pulse wave velocity (PWV_CF_) represents the speed at which the pressure wave travels along the aorta, while the augmentation index (AI) represents the relative contribution of the wave reflections to the amplitude of the aortic pulse wave (pulse pressure) (Laurent et al., 2007). Several CVD risk factors such as hypertension, dyslipidemia, and smoking may lead to aortic stiffness. Aortic stiffness has been found to be associated with future CVD morbidity and mortality (Vlachopoulos et al., 2010; Ben-Shlomo et al., 2014).

The use of PPG for CVD risk assessment has gained popularity due to its simplicity and user-friendliness (Muhajir et al., 2018; Prabhakar, 2019). It signifies peripheral vascular function. PPG measures blood volume changes in the peripheral arterial bed *via* optical techniques in accordance with the contraction and relaxation of the heart. PPG values are affected by several parameters, such as arterial stiffness, resistance, pressure, and blood viscosity (Aminuddin et al., 2018). There are several PPG indexes that have been introduced for the assessment of vascular function, such as stiffness index, reflection index, aging index, and second derivative of PPG (Allen, 2007). Recently, the PPGF index was introduced and proposed to represent vascular age or for vascular function assessment (Aminuddin et al., 2016). The PPGF index was developed by comparing a PPG wave of a subject with the reference who was a 19-year-old healthy individual (Muhajir et al., 2018). The signal variability and repeatability were tested and found to be established as a reliable physiological assessment method (Chellappan, 2010).

Several studies showed that aerobic exercise reduced arterial stiffness (Hayashi et al., 2005; Yoshizawa et al., 2009), while another review found no reduction (Pierce, 2017). This may depend on the age, intensity, and the risk factors present. In addition, a recent study found that the sites of arterial measurement for stiffness may also be influenced, and this was confounded by age. For example, in adults, regular physical activity (PA) was independently associated with lower left and right common carotid artery stiffness and the associated reduction became higher with the increasing age. However, PA was not associated with PWV_CF_ and PWV carotid radial (PWV_CR_) (Zocalo et al., 2022).

PPG is used to determine the effects of acute aerobic exercise on vascular aging (Rozi et al., 2010). The study found that the aging index reduced after 10 min of bicycle exercise among 11 healthy subjects. Another study by Wong and Zhang (2004) found that 4 min of treadmill exercise reduced pulse transit time (PTT)-foot but no change was observed for PTT-peak. In another study, Joseph et al. (2018) found that the c-d-e component of the second derivative became smoother after acute aerobic exercise. So far, limited studies have been conducted to investigate the effects of chronic aerobic exercise on PPG.

The current study was conducted to investigate the effects of pedometer-based exercise among young sedentary men on markers of vascular function. The assessment of vascular functions due to walking may expand the knowledge about the benefit of exercise toward the CVD risk.

## 2 Methodology

### 2.1 Subjects

The study was approved by the Research and Ethics Committee of Universiti Kebangsaan, Malaysia. All measurements were conducted in Universiti Kebangsaan Malaysia Medical Center, Cheras. Subjects were recruited from Institute of Vocational Skills for Youth (IKBN Hulu Langat). The subjects were young men aged 20–40 years with at least two or more CVD risk factors and were randomly divided into the pedometer group (PG, N = 35) and control group (CG, N = 35). The sample size calculation was 66 people, where one group contained 33 people (Chan, 2003).

Total sample size:

$$N=c+\frac{2}{\delta^{2}},$$

where $\delta =\frac{\mu 2-\mu 1}{\sigma }$ .

Here, δ = standardized effect size,

µ1 = mean pre- and post-difference of the intervention group*,

µ2 = mean pre- and post- difference of the control group *, and

σ = typical standard deviation*,

with c = 7.9 for 80% power.

Calculation, c = 7.9,

μ2-μ1 = 0.7 mmHg,

*Values were based on the CRP level that need the highest sample size and obtained from the study that had been conducted by the previous researcher with more or less the same topic and the results of the study were significant (Gray et al., 2009).

All subjects selected were sedentary, which was defined as having daily steps < 5,000 steps. Exclusion criteria were those with chronic diseases such as diabetes mellitus, CVD, inflammatory disease, peripheral vascular disease, lung disease, and liver disease.

### 2.2 Definition of CVD risk factors

Hypertension was defined as systolic blood pressure ≥ 140 and/or diastolic blood pressure ≥ 90 mmHg or on antihypertensive medication (Bakris et al., 2019). Diabetes mellitus was defined as fasting plasma glucose ≥ 7 mmol/L (Alberti and Zimmet, 1998). Smokers were defined as individuals with a habit of daily smoking continued at the time of recruitment for the study (Chin et al., 2012). Abdominal obesity was defined as waist circumference >90 cm (Tan et al., 2004). Family history (FH) of premature CAD was defined as when parents had CAD at <55 (father) or <65 (mother) years of age (Stone et al., 2005). Dyslipidemia was defined as TC > 6.2 mmol/L, TG > 1.7 mmol/L, LDL > 4.2 mmol/L, or HDL < 1.04 mmol/L (Stone et al., 2005).

### 2.3 Exercise intervention

Initially, the PG groups had to increase their daily steps gradually to a minimum of 8,000 steps/day in 4 weeks’ time. Then, they underwent a 12-week pedometer-based walking. The number of steps included from wake-up to bed time every day (five days per week) and was recorded in a standardized diary book. Subjects belonged to the CG were asked to maintain their habitual lifestyle and not to change their daily activity during this program. The parameter assessments were conducted at baseline and after 6-week and 12-week intervention (Omara et al., 2016).

### 2.4 Parameter measurement

#### 2.4.1 Measurement of body anthropometry

Height was measured using a wall-mounted stadiometer (SECA, Hamburg, Germany), and weight was measured using a digital scale (SECA, Hamburg, Germany). Body mass index was then calculated as weight (kg)/height^2^ (m^2^).

#### 2.4.2 Measurement of blood parameters

A volume of 5 ml of blood was withdrawn from the antecubital vein after fasting for a minimum of 8 h. Blood samples were analyzed by Gribbles Pathology Laboratory (Selangor, Malaysia). Serum TG, HDL cholesterol, and TC were measured by using enzymatic methods (Advia 2400 Chemistry Analyzer, Siemens, Tokyo, Japan). The blood glucose was measured by using the enzymatic method using hexokinase and glucose-6-phosphate dehydrogenase enzymes (Advia 2400 Chemistry Analyzer, Siemens, Tokyo, Japan). High-sensitivity C-reactive protein (hs-CRP) was measured *via* particle-enhanced turbidimetric assay. This laboratory obtained the approval of International Organization of Standardization (ISO: MS ISO 15189) in compliance with the standard quality.

#### 2.4.3 Augmentation index (AI)

AI was measured *via* the Vicorder system (SMT Medical, Wuerzburg, Germany). A blood pressure cuff was placed on to the right arm of the subject, which allowed the measurements of the brachial systolic and diastolic BP (SBP/DBP) *via* the oscillometric method and recording of the brachial waveforms. The brachial waveforms were then transformed to the aortic pressure waveforms by using a brachial-to-aortic generalized transfer function. AI was measured by analyzing the aortic pressure waveform using the following formula: augmentation pressure (AP)/pulse pressure x 100 (Laurent et al., 2007).

#### 2.4.4 Carotid femoral pulse wave velocity (PWV_CF_)

Measurements were obtained using the Vicorder system. The subject laid on the bed elevated at 45°. A blood pressure cuff was positioned around the upper thigh to record the femoral pulse. Another 30-mm partial cuff was placed around the neck at the level of the carotid artery. Both cuffs were inflated to 65 mmHg, and the pressure waveforms from the carotid and femoral were recorded simultaneously for 30 s by using a volume displacement method. The foot-to-foot transit time (∆t), which is the delay between the two recorded waves, was obtained *via* an in-built algorithm. ∆D was defined as the distance from the suprasternal notch to the mid of the thigh cuff as instructed by the company. PWV_CF_ was then measured using the following formula: ∆D/∆t (m/s).

#### 2.4.5 Measurement of the PPGF index

The measurement was conducted in a quiet room with controlled temperature between 20 and 25°C. The subject was in the supine position. After resting for 5 min, the PPG probe was attached to the left index finger of the subject, and measurement was performed for 120 s. The signals were acquired through the serial port of pulse-oximeter modules (NiVaRiX 1.0, UKM). The system was connected to a personal computer running the application developed by the manufacturer of NiVaRiX 1.0. The sampling rate was 100 Hz, and the resolution was 16 bits. The PPGF index was derived from an AC component of the PPG morphological changes and compared with a 19-year-old healthy reference (gender-specific) who was identified from the population. The details of the formula could be found in the previous study (Aminuddin et al., 2018).

### 2.5 Statistical analysis

The normality of the data was determined based on inspection of the histogram (plotted as the distribution frequencies) with the acceptable level of skewness (-1 to 1) and kurtosis (-1 to 1). All the data were normally distributed. The unpaired t-test or chi-squared test was used to compare quantitative data or qualitative data for baseline parameters between groups. Pearson’s correlation was conducted to determine the associations between PPGF index and other relevant parameters at baseline. The differences in all the parameters between groups after the intervention were compared using the general linear model (GLM) for repeated measures. A *p*-value of <0.05 was considered significant.

## 3 Results

All subjects in the PG and CG completed the intervention program. The baseline characteristics of the PG and CG are shown in Table 1. No significant differences were observed between the groups for all the parameters at baseline measurements. Table 2 shows the numbers of steps/day in both groups for pre- and post-intervention. Correlation analysis revealed that the PPGF index was correlated significantly with height (*r* = 0.37), systolic BP (SBP) (*r* = -0.32), heart rate (*r* = -0.30), and PWV (*r* = -0.34). Log hs-CRP was associated significantly with AI (*r* = 0.26) (data not shown). After the intervention, there was a significant reduction in BMI, resting BP, resting HR, PWV, AI, and hs-CRP and increase in the PPGF index in the PG when compared to CG, and most of the changes were observed as early as 6 weeks and continued to improve by 12th week (Table 3; Table 4).

**TABLE 1:** Baseline characteristics of the subjects.

Parameter	PG (N = 36)	CG (N = 34)	*p*-value
Age (years)	26.17 ± 6.68	26.62 ± 7.39	0.94
BMI (kg/m^2^)	26.13 ± 5.99	24.49 ± 4.54	0.20
Waist circumference (cm)	86.56 ± 15.09	83.75 ± 14.01	0.42
Resting SBP (mmHg)	120.22 ± 8.97	122.12 ± 8.23	0.36
Resting DBP (mmHg)	64.70 ± 8.84	67.52 ± 8.31	0.17
Resting HR (bpm)	70.81 ± 12.09	70.32 ± 14.20	0.88
PWV_CF_ (m/s)	7.22 ± 0.83	7.25 ± 0.64	0.80
AI (%)	11.88 ± 6.26	11.89 ± 5.44	0.99
PPGF index (%)	56.31 ± 8.71	59.06 ± 9.66	0.13
Total steps/day	4,996.17 ± 49.09	4,986.50 ± 18 .34	0.25
Smoking	72.22%	76.47%	0.69
Family history of CVD	11.11%	11.76%	0.93
hs-CRP (mg/L)	2.23 ± 2.32	2.49 ± 2.25	0.729

**TABLE 2:** Number of steps per day before and after the intervention in both groups.

	Pedometer group (N = 36)		Control group (N = 34)	
	**Week 1**	**Week 12**	**Week 1**	**Week 12**
**Steps/day**	**4,996 ± 805**	**10,128 ± 511** ^**,**#**^	**4,983 ± 366**	**5,697 ± 407**

Data are presented as mean ± SD;

* *p* < 0.05 (time interaction*group);

** *p* < 0.01 (time interaction*group);

# *p* < 0.05 (time effect).

**TABLE 3:** Changes in BMI, cardiovascular parameters, and CRP during and after the intervention.

Parameter	PG (N = 36)			CG (N = 34)		
	Baseline	6 weeks	12 weeks	Baseline	6 weeks	12 weeks
BMI (kg/m^2^)	26.13 ± 5.99	25.88 ± 5.93**	25.43 ± 5.27**^#^	24.49 ± 4.54	24.56 ± 4.51	24.54 ± 4.57
Resting SBP (mmHG)	120.22 ± 8.97	116.39 ± 9.71*	116.33 ± 9.62**^#^	122.12 ± 8.23	118.62 ± 10.57	118.71 ± 10.63
Resting DBP (mmHG)	64.70 ± 8.84	63.89 ± 8.83	63.83 ± 8.73*	67.52 ± 8.31	67.44 ± 9.69	67.82 ± 6.68
Resting HR (bpm)	70.81 ± 12.09	67.92 ± 12.50	66.89 ± 10.83*	70.32 ± 14.20	71.18 ± 14.33	71.23 ± 12.87
hs-CRP(mg/L)	2.23 ± 2.32	1.44 ± 2.13^*^^#^	0.95 ± 1.58^*^^#^	2.49 ± 2.25	2.55 ± 2.15	2.99 ± 3.40

* *p* < 0.05 (time*group interaction);

**p < 0.01 (time*group interaction);

# *p* < 0.05 (main effect of time).

**TABLE 4:** Changes in central and peripheral vascular markers during and after the intervention.

Parameter	PG (N = 36)			CG (N = 34)		
	Baseline	6 weeks	12 weeks	Baseline	6 weeks	12 weeks
PWV_CF_ (m/s)	7.22 ± 0.83	6.84 ± 1.12*	6.42 ± 0.88* ^#^	7.25 ± 0.64	7.31 ± 0.97	7.34 ± 0.87
AI (%)	11.88 ± 6.26	10.83 ± 4.98*	8.83 ± 3.71**^#^	11.89 ± 5.44	12.97 ± 6.28	13.94 ± 6.94
PPGF index (%)	56.31 ± 8.71	60.99 ± 10.34*	61.94 ± 7.79**^#^	59.06 ± 9.66	60.37 ± 10.0	59.80 ± 8.88

* *p* < 0.05 (time*group interaction);

**p < 0.01 (time*group interaction);

# *p* < 0.05 (main effect of time).

## 4 Discussion

### 4.1 Effects of pedometer-based exercise on PWV and AI

A pedometer is a device that counts the number of steps an individual takes to encourage higher levels of physical activities daily alongside building an optimum lifestyle to increase their quality of health. It is a simple step-counting device, and the price is much cheaper than accelerometers. Thus, pedometers are more cost-effective and simple to be used among the population (O’neill et al., 2017). Although accelerometers are more accurate, pedometers are still a valid tool when the aim is to increase the number of steps and not that the intensity of the activity or the time spent for the activity, which can be assessed by accelerometers (O’neill et al., 2017). Previous studies showed that there are large discrepancies between different brands and models and the pedometer accuracy depends on where the device is placed (waist vs. hip) (Husted and Llewellyn, 2017; Gomez Garsia et al., 2022). Thus, standardizing its use in a research setting is important.

This study showed that a pedometer effectively increases the level of physical activity amongst the pedometer group. This research is an interventional study that is rarely conducted in Malaysia, especially among working individuals, as a joint effort to reduce the risk of CVD. The results of this research concluded that subjects included in the sedentary group before intervention (<5,000 steps per day) have successfully increased their number of steps to match the active group (≥10,000 steps per day), which linked to improved cardiovascular risk factors (Wattanapisit and Thanamee, 2017). This result indicated that this intervention is a successful method to promote daily walking to increase the amount of physical activity, especially at the workplace. Therefore, pedometer could potentially contribute to fulfilling the recommended duration for exercise to reduce cardiovascular risks (Arnett et al., 2019). Physical activity can reduce obesity, dyslipidemia, inflammation, and vascular complications by decreasing oxidative stress while improving endothelial function, thereby decreasing the risk of cardiovascular diseases (Aminuddin et al., 2011). On the other hand, the working environment may play an essential role in promoting health and disease prevention (Pereira et al., 2019; Freak-Poli et al., 2020).

Pulse wave velocity is one of the methods used to determine aortic stiffness as it measures the speed at which the pressure wave travels along the aorta. Although this study found that the value of PWV_CF_ is still within the normal range of less than 10 m/s, however, there was a significant decrease in the subjects in the pedometer group. This also applies to the value of AI which also experienced a significant decrease in the pedometer group from 6 weeks of intervention with a sustained downtrend pattern to the end of the intervention. Increased levels of physical activities through walking in this study have also slowed down the process of losing elasticity of arteries and arterial compliance. In the latest European guidelines, PWV_CF_ remains the gold standard for detecting macrovascular stiffness and a value > 10 m/s implies significant pathological macroangiopathy (Williams et al., 2018). The utility of PWV to detect subclinical atherosclerosis before the development of coronary artery disease underpins its clinical value in stratifying patients for risk reduction therapy (Kim and Kim, 2019).

The changes in the arterial structure caused by arteriosclerosis and aging might contribute to the decrease in arterial elasticity and increase in arterial stiffness. This includes smooth muscle hypertrophy, replacing healthy cells with connective tissues. A recent review showed that moderate physical activity levels can cause changes in the arterial structure in several ways (Saladini and Palatini, 2018). The first and easiest way is that exercise leads to acute increase in arterial pressure and heart rate (HR). These physical changes result in blood vessel stretching, which remodels their structure. Second, vasodilation increases during exercise, which would decrease the resistance of blood flow within blood vessels. Third, the increase in pulsatile force within the aorta while walking also acutely releases nitrite oxide and other vasodilators. These factors relax vascular smooth muscles. Nitrate oxide itself possesses strong anti-mitogenic effects that stop the proliferation of vascular smooth muscles. These changes act on limiting or reversing the vascular age-related dysfunction to arterial stiffness. Both aerobic and resistance exercises ameliorate endothelial dysfunction, an early biological sign of cardiovascular dysfunction, by increasing the bioavailability of nitric oxide (Duca et al., 2019). This study also found that the level of hs-CRP decreased significantly after the pedometer-based exercise. Increased inflammation is usually associated with endothelial dysfunction; thus, decreasing inflammation may help to improve endothelial and vascular functions (Theofilis et al., 2021). In addition, there was also a significant association observed between CRP and AI, which indicates a closed relationship between inflammation and vascular function.

### 4.2 Effects of pedometer-based exercise on the PPGF index

Fingertip PPG is now widely used and gaining popularity as it is safe, non-invasive, and easily applicable (Allen, 2007). In our study, the PPGF values for both groups before intervention are low compared to the study conducted by Chellappan et al. (2008) in which the average percentage for healthy subjects aged 19–35 years is 80.96 ± 9.55 per cent. In this study (two or more CVD risk factors), the PPGF value of the pedometer group and the control group is 56.31% and 59.06%, respectively. In another study by Aminuddin et al. (2016), it was found that the PPGF value was 60.10 ± 8.81% for young male subjects aged 20–40 years with two or more CVD risk factors.

The PPGF value in this study was found to correlate significantly with PWV, a standard measurement to evaluate arterial stiffness. This result was also in accordance with the research conducted by Aminuddin et al. (2016), which found that the PPGF index was negatively correlated with PWV, where the PPGF index increases as PWV decreases. The research by Aminuddin et al. (2016) involving 218 male subjects investigated vascular indicators amongst young male individuals with the increased risk of CVD. It was found that the PPGF index had a significant association with PWV (*r* = - 0.27, *p* < 0.01). Other than PWV, this study found that the PPGF index has a significant negative correlation with SBP and HR. Raised SBP and HR show increased stiffness and peripheral arterial resistance, indicative of deteriorated vascular health.

Limited studies have been carried out on the chronic effects of exercise on the PPG wave previously. Madhura and Sandhya (2012) showed that moderate intensity exercise for 8 weeks reduced the stiffness index and reflection index, measured by the PPG technique. In another study, subjects with moderate and high levels of physical activity had lower mean aging character index (ACI) values than those with the low level of physical activity (Wang et al., 2012). ACI was obtained from the analysis of the PPG wave. Regarding PPGF, to the best of our knowledge, this was the first study to elicit the effect of chronic aerobic exercise. The increase in the PPGF index may be due to several reasons. First, it may be due to improvements in arterial elasticity as evidenced by decreased PWV after the intervention. Second, improvement in BP may also increase the PPGF index. These two reasons were derived based on the significant association between PPGF index, PWV, and BP.

## 5 Conclusion

In conclusion, this pedometer-based walking exercise program that has been specifically designed for the working group has successfully proven its effectiveness to reduce CVD risk factors which can be seen as early as 6 weeks after intervention. Walking exercise also managed to improve the central and peripheral vascular functions as evidenced by the reduction in PWV and AI and increase in the PPGF index. Pedometer-based exercise should be encouraged among young working subjects with CVD risk factors.

## 6 Limitations to the study

First, this research only comprises young male subjects, which cannot represent the more significant population of younger and older generation people or female subjects. Second, no measurements were conducted to assess their physical activity, diet, or sleep pattern during intervention in both groups, which may affect the finding. Third, this research only focused on pedometer-based exercise. Future studies should be conducted to observe vascular function changes in various other conditions.

## Data Availability

The original contributions presented in the study are included in the article, further inquiries can be directed to amilia@ppukm.ukm.edu.my.
